# Influenza vaccine effectiveness against detected infection in the community, France, October 2024 to February 2025

**DOI:** 10.2807/1560-7917.ES.2025.30.7.2500074

**Published:** 2025-02-20

**Authors:** François Blanquart, Vincent Vieillefond, Benoit Visseaux, Claire Nour Abou Chakra, Marta C Nunes, Alexandra Jacques, Stephanie Haim-Boukobza, Laurence Josset, Valentin Wehrle, Guillaume Deleglise, Thomas Duret, Marie Anne Rameix-Welti, Bruno Lina, Vincent Enouf, on behalf of the RELAB study group, Antonin Bal

**Affiliations:** 1 Center for Interdisciplinary Research in Biology (CIRB), Collège de France, CNRS, INSERM, PSL Research University, Paris, France; 2 BPO-BIOEPINE-Biogroup, Levallois-Perret, France; 3 Département d’infectiologie, Laboratoire Cerba, Cerba Healthcare, Frépillon, France; 4 Center of Excellence in Respiratory Pathogens (CERP), Hospices Civils de Lyon (HCL), Lyon, France; Centre International de Recherche en Infectiologie (CIRI), Équipe Santé Publique, Épidémiologie et Écologie Évolutive des Maladies Infectieuses (PHE3ID), Inserm U1111, CNRS UMR5308, ENS de Lyon, Université Claude Bernard Lyon 1, Lyon, France; 5 South African Medical Research Council, Vaccines & Infectious Diseases Analytics Research Unit, Faculty of Health Sciences, University of the Witwatersrand, Johannesburg, South Africa; 6 Biogroup Lorraine, Grand Est, France; 7 Laboratoires Cerballiance, Cerba Healthcare, Issy-les-Moulineaux, France; 8 Hospices Civils de Lyon (HCL), Centre National de Référence des virus des infections respiratoires, Institut des Agents Infectieux, Laboratoire de Virologie, Lyon, France; 9 Centre International de Recherche en Infectiologie (CIRI), Laboratoire VirPath, Inserm U1111, CNRS UMR5308, ENS de Lyon, Université Claude Bernard Lyon 1, Lyon, France; 10 BioLBS, 3 Place Félix Faure, 76170 Lillebonne, France; 11 INOVIE GEN-BIO, 8 rue Jacqueline Auriol, 63100 Clermont-Ferrand, France; 12 Molecular Mechanisms of Multiplication of Pneumovirus, Université Versailles St-Quentin en Yvelines, Paris-Saclay INSERM UMR 1173 (2I), Assistance Publique des Hôpitaux de Paris, Paris, France; 13 National Reference Center for Respiratory Viruses, Molecular Mechanisms of Multiplication of Pneumovirus, Institut Pasteur, Université Paris Cité, Paris, France; 14 Members of the RELAB study group are acknowledged at the end of the article

**Keywords:** influenza, vaccine effectiveness, acute respiratory infection, surveillance

## Abstract

Influenza circulates at high levels in Europe since November 2024. Using a test-negative study based on data from French community laboratories between October 2024 and February 2025, we estimated vaccine effectiveness (VE) against PCR-detected influenza infection (44,420/15,052; positive/negative individuals). For all age groups, the overall VE was 42% (95% CI: 37–46%), with 26% (95% CI: 18–34%) against influenza A and 75% (95% CI: 66–82%) against influenza B. Among individuals ≥ 65-year-olds VE was 22% (95% CI: 13–30%) and among 0–64-year-olds, 60% (95% CI: 56–65%).

The incidence of influenza has steeply risen in France and in Europe since November 2024, with, at the end of 2024, a high PCR-test positivity rate for influenza virus and elevated numbers of primary care consultations, as well as increased hospital and intensive care unit (ICU) admissions due to this illness [1-5]. In the United Kingdom (UK), influenza hospital and ICU admissions were in early January 2025 twice as frequent as in the peak of the 2023/24 season for this disease [4]. This interim report provides an overview of vaccine effectiveness (VE) estimates in France based on data from community laboratories. The VE overall and by virus type is presented, as well as among older adults, a high-priority group for vaccination.

## Laboratories, procedures, data collection, and study population

The VE study was conducted using data from RELAB, a network of community-based laboratories located at over 1,600 sites nationwide [6]. All samples from patients presenting in RELAB laboratories for respiratory virus testing, are systematically subjected to a triplex reverse-transcription PCR (RT-PCR) for severe acute respiratory syndrome coronavirus 2 (SARS-CoV-2), influenza, and respiratory syncytial virus. The data collected include sex (male/female), age, presence of symptoms (fever ≥ 38˚C, respiratory symptoms), self-reported vaccination status, PCR technique and RT-PCR testing result.

Participating laboratories weekly transmit virological (RT-PCR results along with quantification cycle (Cq) values, influenza virus typing data when available) and clinical information on all tested patients to the national reference centre (NRC) for respiratory viruses. In this investigation, additional viral genomic sequencing was performed by the NRC on a random subset of study samples testing positive for influenza virus.

The study population included individuals presenting in a RELAB laboratory from week 44 of 2024 to week 5 of 2025 (28/10/2024 to 02/02/2025). Overall, 48,028 of 59,472 patients (81%) presented fever and/or respiratory symptoms. 

## Influenza infection in the community

**Figure 1:**
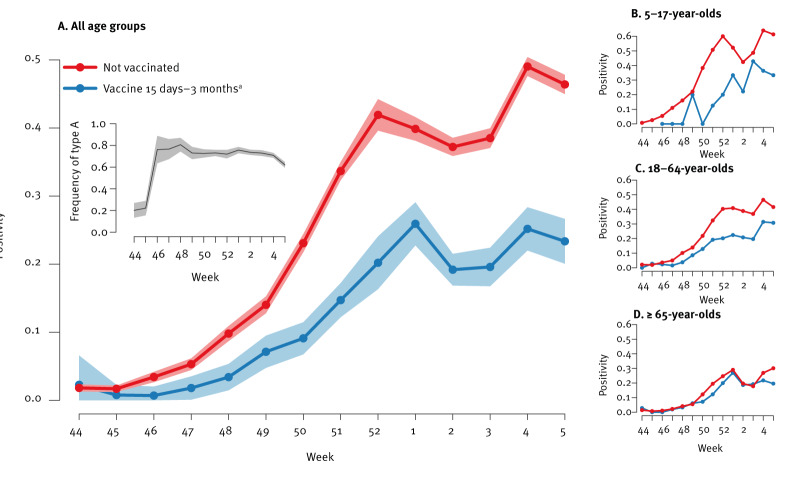
(A) Influenza test positivity per week, for non-vaccinated individuals and individuals vaccinated this season (15 days–3 months prior), (B, C, D) influenza positivity stratified by age group, France, October 2024–February 2025 (n = 59,472)

^a^ Study participants were considered vaccinated if they self-reported receiving an influenza vaccine at least 15 days before getting tested, but no longer than 3 months prior.

Based on analysis of the study samples, influenza circulation in the community in the 2024/25 season started in mid-November (week 46) in non-vaccinated patients (Figure 1). Positivity rate in non-vaccinated patients reached 49% (2,346/4,785) in week 4 of 2025 (vs 25% in vaccinated patients, 173/687). Patients testing negative (n = 44,420), and positive (n = 15,052) for influenza had a slight sex ratio difference, but fever and respiratory symptoms were more common among those testing positive (Pearson’s chi-square test; p < 0.001) (Table 1). The main age group was 18–64 years for both positive and negative patients. Influenza type A represented 72% of all typed viruses (n = 5,841/8,151) with no significant variation since week 50 (Figure 1). Sequencing performed on a random subset of 423 influenza A samples found that A(H1N1)pdm09 was the main A sub-type detected in this population (212/423; 50%), mainly represented by 5a.2a clade with C.1.9.3 being the most detected subclade. A(H3N2) viruses predominantly belonged to 2a.3a.1 - J.2.a subclade while B viruses were mostly represented by V1.1.3a.2-C.5.1 and C.5.7 subclades. Of note, the vaccine composition for 2024/25 northern hemisphere includes 5a.2a.1, 2a.3a.1 and V1.1.3a.2 strains for A(H1N1)pdm09, A(H3N2) and B viruses, respectively [7].

Shaded areas represent 95% confidence intervals. The inset shows the proportion of type A influenza over time. The age group of 0–4 years is not shown because very few individuals from that age class were vaccinated.

**Table 1:** Characteristics of the study population by negative or positive influenza test result, France, October 2024–February 2025 (n = 59,472)

Characteristic	Influenza test result				p value^a^
	Negative, n = 44,420		Positive, n = 15,052		
	Number	%	Number	%	
Sex					
Female	26,754	60	8,702	58	p < 0.001
Male	17,666	40	6,350	42	
Age group in years					
0–4	2,496	5.6	1,729	11	p < 0.001
5–17	3,507	7.9	2,522	17	
18–64	25,126	57	8,727	58	
≥ 65	13,291	30	2,074	14	
Fever (body temperature ≥ 38˚C)					
No	19,281	43	2,247	15	p < 0.001
Yes	19,546	44	11,407	76	
Missing	5,593	13	1,398	9.3	
Respiratory symptom					
No	12,057	27	2,287	15	**p < 0.001**
Yes	29,073	65	11,584	77	
Missing	3,290	7.4	1,181	7.8	
Any symptom					
Any symptom	34,114	77	13,914	92	p < 0.001
Vaccination^b^					
No vaccine	33,545	76	13,136	87	**p < 0.001**
Vaccine < 15 days	1,077	2.4	94	0.6	
Vaccine 15 days–3 months^c^	5,626	13	1,129	7.5	
Vaccine 3–6 months^c^	1,446	3.3	363	2.4	
Vaccine more than 6 months^c^	2,726	6.1	330	2.2	
Year – week of testing					
2024 – 44	3,111	7	52	0.3	**p < 0.001**
2024 – 45	3,582	8.1	55	0.4	
2024 – 46	2,669	6	79	0.5	
2024 – 47	3,030	6.8	149	1	
2024 – 48	3,003	6.8	285	1.9	
2024 – 49	3,174	7.1	461	3.1	
2024 – 50	3,609	8.1	963	6.4	
2024 – 51	3,974	8.9	1,725	11	
2024 – 52	1,414	3.2	836	5.6	
2025 – 1	2,510	5.7	1,437	9.5	
2025 – 2	4,273	9.6	2,134	14	
2025 – 3	3,522	7.9	1,847	12	
2025 – 4	3,285	7.4	2,637	18	
2025 – 5	3,264	7.3	2,392	16	
Influenza type					
A	NA	NA	5,806	39	NA
A+B	NA	NA	35	0.2	NA
B	NA	NA	2,310	15	NA
Subtype – clade					
A(H3N2) – 3C.2a1b.2a.2a.3a.1	NA	NA	84	0.6	NA
A(H1N1)pdm09 – 6B.1A.5a.2a.1	NA	NA	17	0.1	NA
A(H1N1)pdm09 – 6B.1A.5a.2a	NA	NA	195	1.3	NA
B – V1A.3a.2	NA	NA	127	0.8	NA
Missing	NA	NA	14,629	97	NA

NA: not applicable.

^a^ Differences between negative and positive tests were tested with Pearson’s chi-square test and considered significant at the < 0.001 level

^b^ Vaccination is self-reported.

^c^ The time intervals presented in the table are not mutually exclusive and reflect the way participants were asked to self-report since when they were last vaccinated against influenza.

### Vaccine effectiveness by influenza type

As the vaccination campaign in France started in mid-October, participants were unlikely to have received vaccination 3 to 6 months prior to the study period when they were tested (see Discussion). Study participants were considered vaccinated if they self-reported receiving an influenza vaccine at least 15 days before getting tested, but no longer than 3 months prior. Over the whole period, vaccine coverage among PCR-tested individuals was 6.5% in 18–64-year-olds and 36% in ≥ 65-year-olds (Table 2), and increased over the season in the overall population from 1.8% (44/2,481) in week 44 to 19% (396/2,117) in week 52 of 2024, and up to 52% (266/515) in the ≥ 65-year-olds. We used a test-negative design to infer VE against detected influenza infection. We fitted a logistic (binomial) linear model to test result (negative/positive), as a function of sex, age category, PCR technique, week, and vaccination status, age and week being the most important confounders. We tested several models with week as a continuous or categorical factor, and including or not an interaction between week and vaccine status (which can be significant if effectiveness changes over time). We retained the best model in terms of the Akaike Information Criterion. The retained model included week as a categorical factor and no interaction, and we used this model in subsequent analyses. The VE was measured as the odds ratio (OR) of the vaccine effect on positivity (the exponential of the inferred coefficient corresponding to vaccine status in the linear model). When test positivity is small enough, ORs approximate the risk ratio [8].

**Table 2:** Vaccination status by age group in a subset of the study population, France, October 2024–February 2025 (n = 54,607)^a^

Vaccine	Age groups in years							
	18–64, n = 31,856		0–4 n = 4,191		5–17 n = 5,929		≥ 65 n = 12,631	
	Number	%	Number	%	Number	%	Number	%
No vaccine	29,359	92	4,148	99	5,839	98	7,335	58
Vaccine < 15 days	416	1.3	9	0.2	19	0.3	727	5.8
Vaccine 15 days–3 months	2,081	6.5	34	0.8	71	1.2	4,569	36

^a^ People vaccinated over 3 months prior to testing (n = 4,865) are not considered in the table.

**Figure 2:**
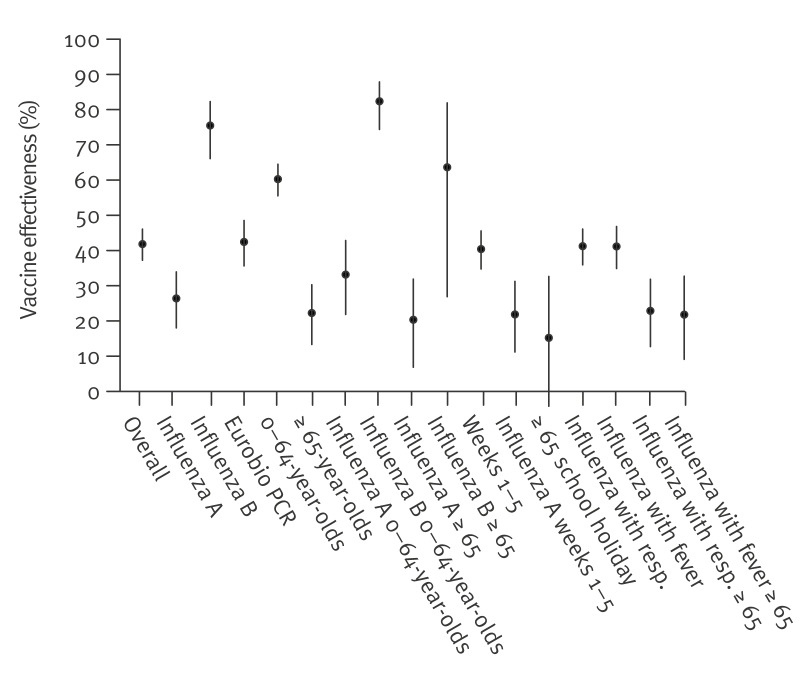
Vaccine effectiveness against influenza overall, and stratified by virus type and/or characteristics of individuals in the study, as well as overall vaccine effectiveness in a subset of participants tested with a single kind of PCR assay^a^, France, October 2024–February 2025 (n = 59,472)

^a^ The PCR assay in question was Eurobioplex FluCoSyn ebx-042, Eurobio Scientific, Les Ulys, France.

The resulting VE estimates for individuals vaccinated 15 days–3 months before testing was 42% (95% CI: 37 to 46%) for influenza overall, 26% (95% CI: 18 to 34%) for influenza A, and 75% (95% CI: 66 to 82%) for influenza B infections (Figure 2, Table 3).

Points are the maximum likelihood estimates, segments show the 95% confidence intervals. All numbers are presented in Table 3. From left to right in the graph, vaccine effectiveness is presented overall, against influenza A, influenza B, in the subset of tests done with the Eurobio PCR technique, in the 0–64-year-olds, in the ≥ 65-year-olds, against influenza A in the 0–64-year-olds, against influenza B in the 0–64-year-olds, against influenza A in the ≥ 65-year-olds, against influenza B in the ≥ 65-year-olds, at weeks 1–5, against influenza A at weeks 1–5, in the subset of ≥ 65-year-olds during the school holidays (weeks 52–1), in the subset of patients presenting with respiratory symptoms, the subset of patients presenting with fever, the subset of ≥ 65-year-olds presenting with respiratory symptoms, and the subset of ≥ 65-year-olds presenting with fever.

**Table 3:** Vaccine effectiveness against influenza overall, and stratified by virus type and/or characteristics of individuals in the study, as well as overall vaccine effectiveness in a subset of participants tested with a single kind of PCR assay^a^, France, October 2024–February 2025 (n = 59,472)

Analysis	Cases		Controls		VE in % (95% CI)
	All	Vaccinated	All	Vaccinated	
Overall	15,052	1,129	44,420	5,626	42 (37 to 46)
Influenza A	5,806	555	46,730	5,666	26 (18 to 34)
Influenza B	2,310	40	50,226	6,181	75 (66 to 82)
Eurobio PCR	6,775	503	23,149	2,912	42 (36 to 49)
0–64-year-olds	12,978	418	31,129	1,768	60 (56 to 64)
≥ 65-year-olds	2,074	711	13,291	3,858	22 (13 to 30)
Influenza A 0–64-year-olds	4,801	208	33,391	1,797	33 (22 to 43)
Influenza B 0–64-year-olds	2,262	29	35,930	1,976	82 (74 to 88)
Influenza A ≥ 65-year-olds	1,005	347	13,339	3,869	20 (6.9 to 32)
Influenza B ≥ 65-year-olds	48	11	14,296	4,205	64 (27 to 82)
Weeks 1–5	10,447	842	16,854	2,937	40 (35 to 46)
Influenza A weeks 1–5	4,020	429	18,527	2,969	22 (11 to 31)
≥ 65-year-olds school holiday	466	192	1,321	589	15 (−6.7 to 33)
Influenza with respiratory symptoms	11,584	944	29,073	3,822	41 (36 to 46)
Influenza with fever	11,407	745	19,546	2,006	41 (35 to 47)
Influenza with respiratory symptoms ≥ 65-year-olds	1,657	600	8,546	2,620	23 (13 to 32)
Influenza with fever ≥ 65-year-olds	1,260	445	4,278	1,267	22 (9.1 to 33)

^a^ The PCR assay in question was Eurobioplex FluCoSyn ebx-042, Eurobio Scientific, Les Ulys, France.

When focusing on results from a single type of PCR test (the most frequently used in RELAB laboratories, Eurobioplex FluCoSyn ebx-042, Eurobio Scientific, Les Ulys, France), the overall VE was 42% (95% CI: 36 to 49%) suggesting that overall results were robust. Regardless of the assay used, VE was estimated at 60% (95% CI: 56 to 64%) among the 0–64-year-olds and 22% (95% CI: 13 to 30%) among those aged ≥ 65 years. As type B influenza circulates preferentially in younger age groups, as shown in Supplementary Figure 1, we calculated effectiveness by age and type and found 33% (95% CI: 22 to 43%) vs 20% (95% CI: 6.9 to 32%) against influenza A in 0–64 and ≥ 65 years old, and 82% (95% CI: 74 to 88%) vs 64% (95% CI: 27 to 82%) against influenza B in 0–64 and ≥ 65-year-olds. The VE did not change over time: it was comparable at the end of the period (weeks 1–5) at 40% (95% CI: 35 to 46%) over all influenza viruses, and 22% (95% CI: 11 to 31%) for influenza A. We examined whether effectiveness was particularly low during the end-of-year/school holiday festive period, as vaccination could have been associated with more contacts during that period (if individuals vaccinate when they plan to have more contacts). The VE was slightly lower among individuals ≥ 65 years in the weeks of school holiday (weeks 52–1), with largely overlapping CIs (VE: 15%; 95% CI: −6.7 to 33%). Finally, results were also robust when focusing on symptomatic individuals, with VE at 41% (95% CI: 36 to 46%) for the subpopulation presenting with respiratory symptoms and 41% (95% CI: 35 to 47%) for the subpopulation presenting with fever.

## Discussion

In France, the influenza vaccination campaign was launched in week 42 (mid-October) in targeted populations. The vaccines provided were quadrivalent inactivated vaccines, with standard dose, and no adjuvant. No enhanced vaccines were available. Early data presented herein suggest low to high vaccine effectiveness (VE) against influenza types A and B in the community, with estimates of 26% and 75% respectively.

These interim VE estimates by influenza type are consistent with trends observed during the 2022/23 season in Europe and during the 2023/24 season in the United States where VE against influenza B was higher than that against A viruses [9,10]. In both Europe and France, estimated VE against type A for 2023/24 was 51% in primary care, which is higher than in the current season [11,12]. In 2024/25, despite the mismatch between the clade of the predominant circulating A(H1N1)pdm09 strains (5a.2a) and the one in the vaccine strain (5a.2a.1), A(H1N1)pdm09 circulating strains were antigenically similar to the A/Victoria/4897/2022 virus included in the 2024/25 northern hemisphere vaccine, while some A(H3N2) isolates were antigenically distinct from the vaccine strain [3]. Studies focusing on VE by subtype are needed to assess the role of A(H3N2) viruses, which are known to undergo substantial genetic drift and to present antigenic variation [13], on the reduced VE observed herein. A recent study conducted in medically-attended outpatients in the United States during the 2023/24 season showed a similar VE of about 30% for both A(H1N1)pdm09 and A(H3N2) subtypes [9] while a Canadian study conducted in 2024/25 season in outpatients found 53% and 54% VE for A(H1N1)pdm09 and A(H3N2), respectively [14]. 

In our current study in the 2024/25 season so far, the VE was inferred to be lower in  ≥ 65 year-olds (22%) than in 0–64-year-olds (60%), highlighting the need to consider targeted immunisation strategies, such as the high-dose (available during 2023/24 season in France) or adjuvanted vaccines which were not available this season in France [15]. In the 2024/25 Canadian study, where high-dose and adjuvanted vaccines were used, estimated VE against influenza at 59% in ≥ 65-year-old patients and at 54% in 1–64-year-olds [14]. Besides vaccine formulation, other factors, such as the intensity of the epidemic, the vaccine coverage, the circulation of a viral escape variant, or childhood immune imprinting [16], might also contribute to the differences in VE observed between older adults in the two countries. These might, however, also be caused by behavioural differences between vaccinated and unvaccinated individuals. On the other hand, the reduced VE in our study in older adults is consistent with previous reports that have documented lower immune responses in this population group due to immunosenescence and comorbidities [17]. 

The current analysis is subject to several limitations. The reason for PCR testing and how it might depend on vaccination status was unknown, which may introduce selection bias. Selection bias happens in observational studies focusing on the population seeking a PCR test, for example when both vaccination and infection encourage test-seeking. Conditioning on the collider (test-seeking behaviour) creates a negative correlation between vaccination and test positivity which would bias effectiveness upwards [18-20]. In our data, the vaccine coverage at week 52 was 52% in ≥ 65-year-old individuals, comparable to the nationwide figure of 50%, suggesting that vaccination did not influence test-seeking [21]. Another concern is confounding by health status (co-morbidities) of vaccinated individuals, which can act in either direction depending on whether healthy individuals (‘healthy vaccine bias’) or conversely individuals with co-morbidities (‘confounding by indication’) are more likely to get vaccinated [22,23]. Such confounding may play a limited role in our study, as we focus on mild symptoms. Self-reported vaccination status is subject to recall bias, but this should not have impacted the present analysis. Notably, around 3% (1,809/59,472) of individuals reported receiving the influenza vaccine 3 to 6 months prior to testing for respiratory infection, 814 of them before mid-January, which was not possible given the timing of the vaccination campaign in France. This small subset, which we excluded from the main analysis, had an estimated VE of 44% (95% CI: 37 to 51%) comparable to that in participants vaccinated in the 15 days–3 months prior to testing. Furthermore, 54% (8,151/15,052) of samples were subtyped and only 3% (423/15,052) were sequenced, restricting our ability to analyse VE for specific subtypes or clades. End-of-season estimates incorporating sequencing data will provide a more comprehensive assessment of VE and identify potential escape variants. The study focused on VE against infection rather than severe disease outcomes, which limits the generalisability to hospitalisation and mortality rates. Given the high mortality observed this season in France, additional hospital-based studies would provide complementary insights and are particularly needed given the inferred low effectiveness in older adults [3]. The low VE observed herein for type A notably in people aged ≥ 65 years could contribute to the high rate of hospitalisations observed in France this season in addition to the low vaccine coverage [3].

## Conclusion

Preliminary findings for the 2024/25 influenza season highlight the continued value of vaccination in reducing influenza burden, with effectiveness varying by virus type and age group. Strengthened public health efforts to increase vaccine uptake and potentially to improve vaccine formulations remain critical to reducing the impact of seasonal influenza.

## Supplementary Data

157000 Supplementary Material
