# Supplementary material for: Influenza vaccine effectiveness against detected infection in the community, France, October 2024 to February 2025
Source: Euro Surveill. 2025 Feb 20;30(7):2500074. doi: 10.2807/1560-7917.ES.2025.30.7.2500074 (PMC11843621; doi:10.2807/1560-7917.ES.2025.30.7.2500074)
Supplement: Supplementary Material [file 2500074_SupplementaryMaterial.pdf]

This supplementary material is hosted by *Eurosurveillance* as supporting information alongside the article **“Influenza vaccine effectiveness against detected infection in the community, France, October 2024 to February 2025”,** on behalf of the authors, who remain responsible for the accuracy and appropriateness of the content. The same standards for ethics, copyright, attributions and permissions as for the article apply. Supplements are not edited by *Eurosurveillance* and the journal is not responsible for the maintenance of any links or email addresses provided therein.

**Supplementary Figure 1:** The frequency of type A influenza among typed positive tests, for the 0-4 years old (A), 5-17 years old (B), 18-64 years old (C) and the $\geq$65 years old (D). The line shows the frequency and the shaded areas the 95% confidence interval.

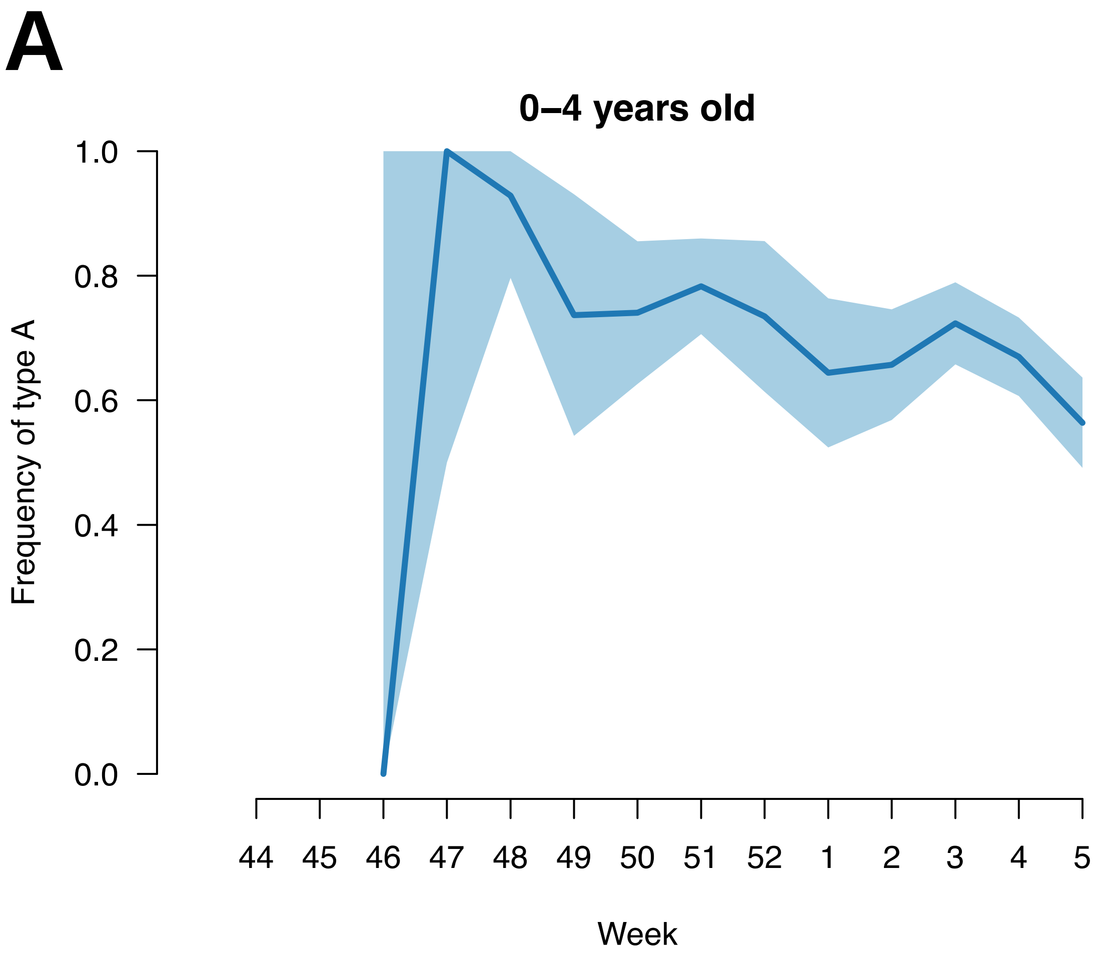

**B**

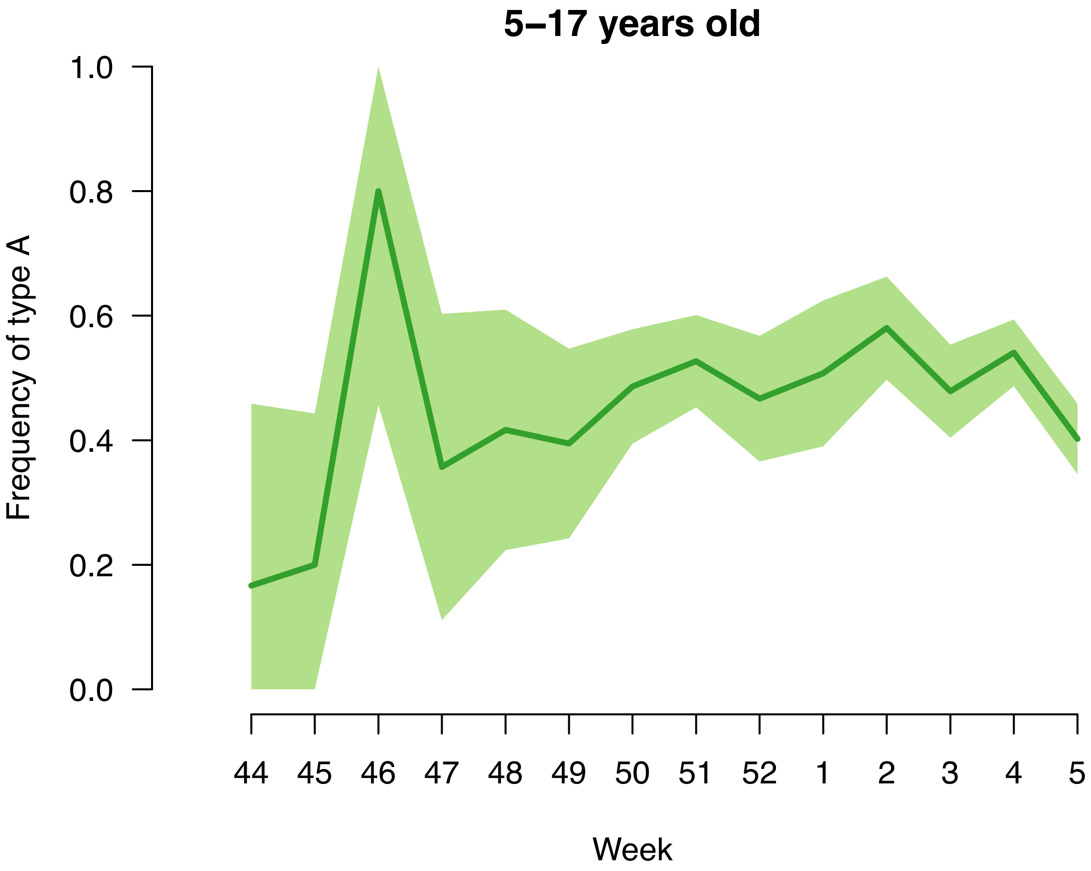
5–17 years old
Frequency of type A
0.0
0.2
0.4
0.6
0.8
1.0
44
45
46
47
48
49
50
51
52
1
2
3
4
5
Week

**C**

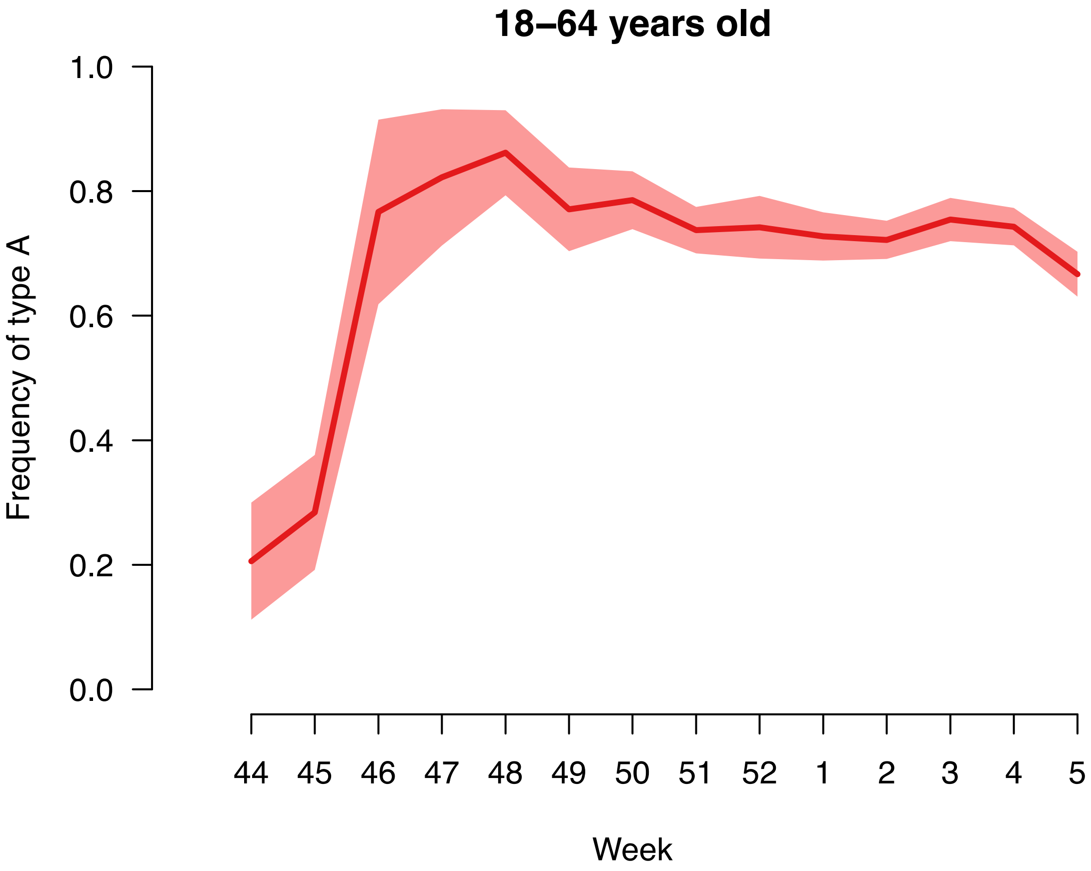
18–64 years old
Frequency of type A
0.0
0.2
0.4
0.6
0.8
1.0
44 45 46 47 48 49 50 51 52 1 2 3 4 5
Week

D

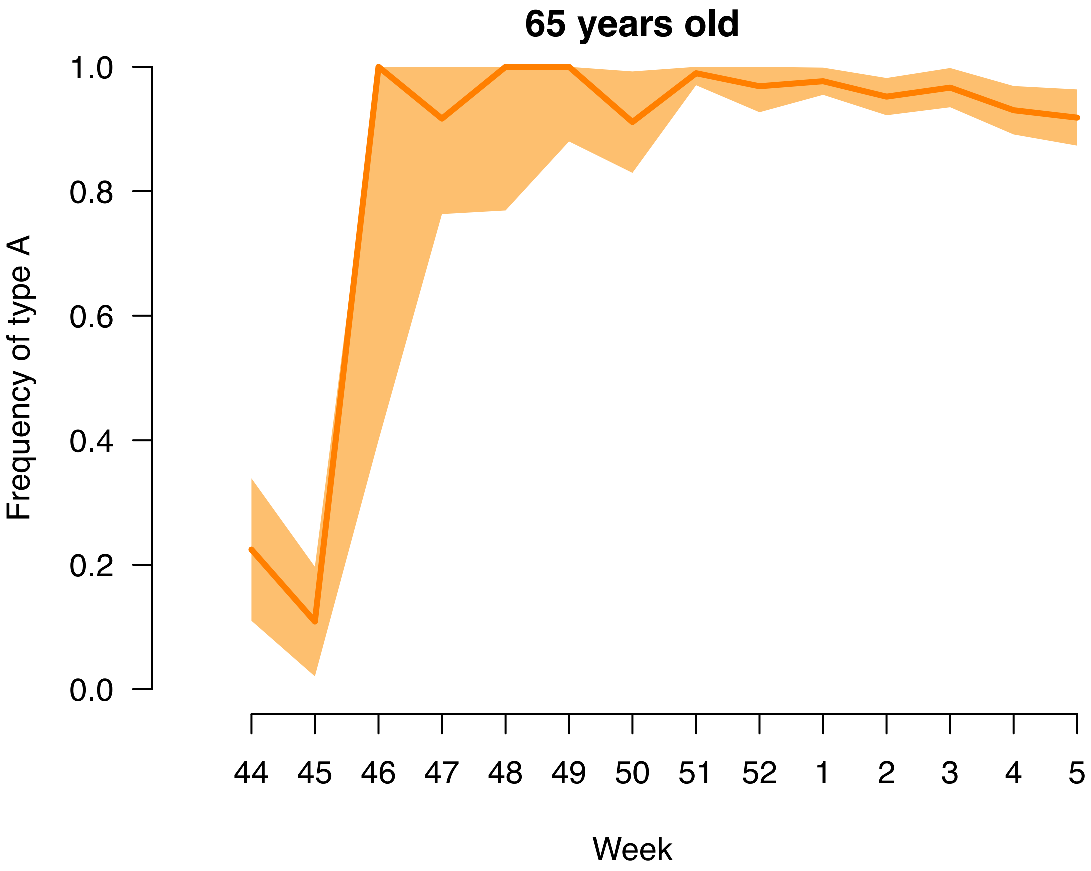
65 years old
Frequency of type A
1.0
0.8
0.6
0.4
0.2
0.0
44
45
46
47
48
49
50
51
52
1
2
3
4
5
Week
